# microRNA and thyroid hormone signaling in cardiac and skeletal muscle

**DOI:** 10.1186/s13578-017-0141-y

**Published:** 2017-03-21

**Authors:** Duo Zhang, Yan Li, Shengnan Liu, Yu-cheng Wang, Feifan Guo, Qiwei Zhai, Jingjing Jiang, Hao Ying

**Affiliations:** 1 Key Laboratory of Food Safety Research, Institute for Nutritional Sciences, Shanghai Institutes for Biological Sciences, Chinese Academy of Sciences, University of Chinese Academy of Sciences, 320 Yueyang Road, Shanghai, 200031 China; 2 Shanghai Clinical Center, Chinese Academy of Sciences, Shanghai Xuhui Central Hospital, 966 Middle Huaihai Road, Shanghai, 200031 China; 3 Key Laboratory of Nutrition and Metabolism, Institute for Nutritional Sciences, Shanghai Institutes for Biological Sciences, Chinese Academy of Sciences, University of Chinese Academy of Sciences, 320 Yueyang Road, Shanghai, 200031 China; 4 Department of Endocrinology and Metabolism, Zhongshan Hospital, Fudan University, 180 Fenglin Road, Shanghai, 200032 China; 5 Key Laboratory of Food Safety Risk Assessment, Ministry of Health, Beijing, China; 6 Shanghai Institutes for Biological Sciences, Chinese Academy of Sciences, Institute for Nutritional Sciences, Room A1912, New Life Science Building, 320 Yueyang Road, Shanghai, 200031 China

**Keywords:** Thyroid hormone, miRNA, Heart, Skeletal muscle

## Abstract

Thyroid hormone (TH) signaling plays critical roles in the differentiation, growth, metabolism, and physiological function of all organs or tissues, including heart and skeletal muscle. Due to the significant progress in our understanding of the molecular mechanisms that underlie TH action, it’s widely accepted that TH signaling is regulated at multiple levels. A growing number of discoveries suggest that microRNAs (miRNAs) act as fine-tune regulators of gene expression and adds sophisticated regulatory tiers to signaling pathways. Recently, some pioneering studies in cardiac and skeletal muscle demonstrating the interplay between miRNAs and TH signaling suggest that miRNAs might mediate and/or modulate TH signaling. This review presents recent advances involving the crosstalk between miRNAs and TH signaling and current evidence showing the importance of miRNA in TH signaling with particular emphasis on the study of muscle-specific miRNAs (myomiRs) in cardiac and skeletal muscle. Although the research of the reciprocal regulation of miRNAs and TH signaling is only at the beginning stage, it has already contributed to our current understanding of both TH action and miRNA biology. We also encourage further investigations to address the relative contributions of miRNAs in TH signaling under physiological and pathological conditions and how a group of miRNAs are coordinated to integrate into the complex hierarchical regulatory network of TH.

## Background

### Thyroid hormone

Thyroid, the largest endocrine organ of human body, synthesizes and secretes thyroid hormones (THs), including triiodothyronine (T3) and thyroxine (T4), which act upon nearly every cell in the body. The production of TH by thyroid is tightly controlled by a negative feedback loop of hypothalamus–pituitary–thyroid axis [1–3]. T4 is the most abundant TH in the blood, which is converted into T3, the biologically active form of TH, by iodothyronine deiodinase selenoenzymes in cells. The physiological roles of TH have been extensively studied for more than a century. It is known that TH plays critical roles in growth, differentiation, development, and metabolism [2, 4–6]. One of the most important questions in the field of TH action is how the diverse biological activities of TH are achieved.

### Thyroid hormone receptor

The action of TH is mediated mainly through its binding to thyroid hormone receptor (TR), which is a ligand-dependent transcription factor that belongs to the nuclear receptor superfamily [3]. TR binds to the thyroid hormone response element (TRE) within the promoter of target genes as either homodimers or heterodimers with retinoid X receptor (RXR). The transcription mediated by TR involves the T3 binding-induced conformational change of TR and the dynamic interplay with nuclear receptor coregulators [1, 3]. In general, unliganded TR interacts with corepressors to inhibit target gene transcription, while, in the presence of T3, T3-bound TR recruits coactivators to promote the transcription [1]. In contrast, the mechanisms underlying T3-mediated transcriptional repression are still poorly understood. TRs are encoded by two genes, TRα and TRβ, which generate multiple isoforms through alternative splicing or promoter. Among these TRs, only TRα1 and TRβ1-3 have the capacity to bind T3 [7]. TRα and TRβ genes are differentially expressed during development and in adult tissues, which provides an additional means to modulate the TH actions in a temporal and spatial manner [3, 7]. In addition, TR is subject to posttranslational modification by phosphorylation and sumoylation, which also contribute to the modulation of TR activity [8, 9].

## Thyroid hormone and its action on cardiac and skeletal muscle

### Effects of thyroid hormone on heart

The close relationship between thyroid and heart was noted in the earliest descriptions of hyperthyroidism. The impact of thyrotoxicosis on cardiovascular system was first described in 1785 by Caleb Parry, a British physician who noticed an association between a swelling neck and heart failure [10]. In general, the profound effects of TH on cardiac function include enhancing overall total protein synthesis, lowering systemic vascular resistance, increasing blood volume, and modulating inotropic and chronotropic response [11]. The combination of these effects on both the circulation and the heart itself results in an increase in cardiac output. Hyperthyroid patients exhibit a high cardiac output state, whereas hypothyroid patients display low cardiac output, decreased stroke volume, decreased vascular volume, and increased systemic vascular resistance [11, 12]. It is well accepted that both the direct regulation of target genes by TH and the indirect effects of TH on hemodynamics contribute to these changes [11–13].

Myosin is one of the key components of contractile apparatus, where chemical energy of adenosine triphosphate (ATP) is converted to mechanical work [14]. There are three isoenzymes of ventricular myosin in heart, which differ in their myosin heavy chain (MHC) α and β composition: myosin V1 (α/α), myosin V2 (α/β), and myosin V3 (β/β) [14]. Myosin V1 has the highest ATPase activity and V3 the lowest. Myosin V2 has an ATPase activity intermediate between V1 and V3. Thus, the ratio of the V1 to the V3 isoenzyme of myosin determines cardiac contractility and correlates with cardiac muscle performance as indicated by mechanical, myothermal, and biological analysis [14]. The relative amounts of the three isoforms (V1, V2, and V3) not only change with age, or stress (exercise or ventricular pressure overload), but also change under altered thyroid states (hypothyroidism or hyperthyroidism) [15].

It is known that TH has opposing effects on α-MHC and β-MHC gene expression [16, 17]. Putative TREs for both α-MHC and β-MHC genes have been reported in the promoter region of these two genes. The two TREs identified in the promoter of α-MHC are imperfect direct repeats, which are separated by four nucleotides [18, 19]. Regarding β-MHC, a putative negative TRE (nTRE) containing a single half-site is found adjacent to the TATA box [20, 21]. The increase of α-MHC gene expression and the decrease of β-MHC gene expression by T3 result in an increase in myosin V1 levels and enhanced cardiac contractility [19]. In contrast, induction of a hypothyroid state increases myosin V3 composition, which leads to a decrease in velocity of fiber shortening [16, 19]. In addition, a surge in TH levels after birth also contributes to the developmental switch in the ventricles of rat, in which α-MHC mRNA increases shortly after birth and almost replaces β-MHC mRNA completely in a week [22]. Although the developmental pattern for MHC isoforms is different and the effect of T3 on α-MHC is small in higher mammals, a clinical study showed that T4 replacement improved cardiac function of a hypothyroid patient, which was accompanied with a more than 10-fold increase of α-MHC mRNA levels in the patient’s ventricles [23]. Therefore, due to its ability to control the expression of MHC isoforms, TH is a critical player in the regulation of cardiac function.

Changing concentrations of cytosolic Ca^2+^ in cardiac myocytes controls each cycle of contraction and relaxation [24]. Cardiac sarcoplasmic reticulum calcium ATPase (SERCA2), which is an intracellular ion pump, plays a critical role in maintaining the intracellular Ca^2+^ homeostasis [25]. SERCA2 removes the Ca^2+^ from the cytosol and stores the Ca^2+^ in the sarcoplasmic reticulum, which leads to diastolic relaxation [25]. The expression of SERCA2 was decreased in hypothyroid rats, while the levels of SERCA2 could be elevated by T3 treatment, suggesting that SERCA2 is positively regulated by TH [26, 27]. Thus, TH is able to relax the heart and enhance cardiac output by reducing the amount of cytosolic Ca^2+^ through increasing SERCA2 expression [26, 27]. For the time being, three TREs arranged as direct repeats (DRs) and inverted palindromes have been identified in the promoter region of SERCA2 gene [28]. Besides regulating SERCA2 expression, T3 can also shape heart function by targeting other ion channels such as voltage-gated potassium (Kv) channel Kv1.5, Na^+^/K^+^-ATPase, and hyperpolarization activated cyclic nucleotide-gated channel [29, 30]. In addition, TH may enhance the cardiac sensitivity to catecholamines through controlling the β-adrenergic receptor expression [31].

Recent findings from TR mutant mouse models substantiate the role of TH in the physiology of heart [32]. Introducing a dominant negative mutant TRβ into the heart of mice suppressed the mRNA expression of α-MHC and SERCA2, but upregulated the mRNA levels of β-MHC, which led to prolonged cardiac muscle contraction and QRS interval [33]. Studies using TR isoform-specific mouse models have elucidated that TRα and TRβ play differential roles in the regulation of heart rate [34]. Mice deficient in TRα showed decreased heart rate and prolonged QRS interval, whereas mice lacking TRβ had elevated heart rate, which was resistant to TH treatment. These findings suggest that TRα1 is more important for maintaining baseline heart rate, whereas TRβ may be only involved in TH-mediated stimulation of heart rate [34, 35]. Due to the beneficial effect of TH on cardiac function, T3 has been employed for heart surgery including cardiac transplantation and cardiac bypass surgery.

### Effects of thyroid hormone on skeletal muscle

Skeletal muscle is a major target organ of TH. Myopathic symptoms are very common among hyperthyroid or hypothyroid patients [36]. The effects of THs on muscle contractility and metabolism have been extensively studied [5]. It is known that MyHC gene expression is controlled by TH [16, 37, 38]. Muscles of hypothyroid patients typically display a conversion from fast to slow fiber types, and a more efficient energy metabolism [39]. Hypothyroid patients have less type-II fibers in the muscle, as compared to euthyroid patients [39]. Interestingly, hypothyroid females have higher proportion of type-II fibers than hypothyroid males; however, type-II fiber atrophy occurs only in hypothyroid female patients [40], suggesting there is a gender-dependent mechanism involved.

In general, experimental hyperthyroidism in rats using T3 could induce a reversible slow-to-fast MyHC isoform transition from I → IIa → IIx → IIb [41]. However, gender- and muscle-specific differences were always observed in the regulation of MyHC isoforms by T3 in rats. Normally, almost all myofibers in the soleus muscle of rats expressed the slow MyHC isoform (type-I fibers) [38]. Four weeks of T3 treatment resulted in an increase of type-IIA fibers and a decrease of type-I fibers [42]. After T3 treatment, compared to male rats, the soleus muscle of female rats expressed more type-I fibers and less type-I/IIa and type-I/IIax fibers [39]. Moreover, the IIx content from the type-I/IIax fibers increased more in T3-treated male rats than that in female rats, while the upregulation of IIa content was greater in female rats than that in male rats [39]. In addition, it has been reported that T3 treatment induced IIx MyHC isoform expression only in the soleus muscle of male rats, whereas IIx expression could not be detected in female rats at any age [43].

In contrast to soleus muscles, extensor digitorum longus (EDL) muscles contain predominantly fast MyHC isoforms (IIa, IIx, and IIb) [44]. Increased levels of MyHC-IIa were observed in the EDL muscle of hypothyroid rat [45]. One study showed that long-term T3 treatment reduced both MyHC-IIb and MyHC-IIa expression at mRNA and protein levels in the EDL muscle [46]. However, in another study, chronic hyperthyroidism increased MyHC-IIb mRNA expression without changing the protein levels in rat EDL muscle [47]. It has been reported that T3 treatment in euthyroid rats resulted in a transition from IIa to IIb fibers only in the EDL muscle of female rats, but not in that of male rats, further suggesting there is a gender difference in fiber-type conversion [40]. These studies indicate that the effect of TH on muscle contractibility, endurance, and the response to fatigue might differ between females and males, presumably as a result of a gender difference in the regulation of fiber-type switch.

## MicroRNAs biogenesis and functions

MicroRNAs (miRNAs), first identified in *C. elegans* in the early 1900s [48], are a group of highly conserved noncoding RNAs and approximately 22 nucleotides in length [49]. miRNAs function primarily as negative regulators of gene expression at the post-transcriptional level [49, 50]. miRNAs can be classified as intronic and intergenic based on their genomic location [49, 51]. In general, intronic miRNAs are located in the introns of protein coding genes and transcribed along with their host genes, while intergenic miRNAs have their own promoters and are transcribed as independent transcripts [49, 51].

miRNAs are usually transcribed by RNA polymerase II as primary miRNAs (pri-miRNAs) in the nucleus [52]. However, it also has been reported that a few miRNAs requires RNA polymerase III to generate pri-miRNAs [53]. After transcription, pri-miRNAs are then processed by microprocessor complex, which is composed of two core components, dsRNA-binding protein DGCR8 and RNase III endonuclease Drosha [52, 54]. Microprocessor complex binds to the stem-loop structure of pri-miRNAs and cleaves the primary transcripts to release a hairpin-shaped RNA molecule known as precursor miRNAs (pre-miRNAs) [52, 54]. The double-stranded pre-miRNAs are 70–100 nucleotides in length, and subsequently transported from nucleus to the cytoplasm by Exportin-5 for further processing [52, 54]. Dicer, which is a RNase III endonuclease and responsible for the maturation of miRNAs in the cytoplasm, cleaves pre-miRNA to generate a double stranded miRNA duplex (miRNA–miRNA*) with 20–25 nucleotides in length [52, 54, 55]. The mature miRNA duplex is then recognized by the RNA induced silencing complex (RISC) containing Dicer and AGO2 (argonaute RISC catalytic component 2). Usually, only one strand of miRNA duplex is preferentially chosen to be incorporated into the RISC to form miRNA-induced silencing complex (miRISC) [55, 56]. The miRNA-loaded RISC binds to the target mRNAs and silences the gene expression through either degradation of mRNA or inhibition of translation [55, 56].

## MiRNA and thyroid hormone signaling

TH signaling has profound effects on many physiologic processes. The effectiveness of TH signaling relies on their capacity to tightly control the expression of target genes in time and space. The temporal and spatial activities of TH could be achieved through the regulation of the systemic and local levels of TH. The circulating TH levels are exquisitely regulated by a negative-feedback system that involves the hypothalamus–pituitary–thyroid axis, while the intracellular TH levels are modulated by type 2 deiodinase, which is responsible for the conversion of prohormone T4 to bioactive T3. Recently, the miRNA field has grown tremendously and emerging evidence suggests that miRNAs not only confer signaling robustness as amplifiers, balancers, or buffers but also play important roles in signaling crosstalk and coordination as nodes of signaling networks. In 2007, Olson’s group published a research article in Science magazine that first linked the role of miRNA to TH signaling [57]. Soon afterward, the possible involvement of miRNAs in TH signaling networks has been indicated by several studies in a variety of model systems. In this review we summarize the major research progress with an emphasis on muscle-specific miRNAs (myomiRs), including miR-208a/b, miR-499, miR-133, which play important roles in the regulation of the development, plasticity, and health of mammalian skeletal and cardiac muscles.

### MiR-208a

MiR-208a is located in the intron of α-MHC gene, which encodes a major cardiac contractile protein [58]. It was shown that cardiac-specific miR-208a was required for cardiomyocyte hypertrophy, fibrosis, and the expression of β-MHC in response to hypothyroidism [57]. Ablation of miR-208a attenuated cardiac hypertrophy in response to pressure overload and reduced β-MHC expression [57]. While cardiac overexpression of miR-208a was sufficient to induce cardiac hypertrophy and cardiac conduction abnormalities [57]. Further study showed that miR-208a acted through repressing the TR co-regulator THRAP1 and myostatin, two negative regulators of muscle growth and hypertrophy [57, 58]. Accordingly, inhibition of miR-208a by antisense oligonucleotide delivery improved cardiac function, overall health, and survival during hypertension-induced heart failure [59].

It is well established that TH promotes α-MHC and inhibits β-MHC expression in the heart [16, 17]. Similar to the regulation of α-MHC by TH, the expression of miR-208a is also significantly blunted in the adult heart in response to hypothyroidism. To further explore the roles of miR-208a in the cardiac muscle, β-MHC expression was investigated in miR-208a knockout mice treated with propylthiouracil (PTU), which rendered the mice hypothyroid [58]. Interestingly, hypothyroidism could not induce β-MHC expression in the heart of miR-208a null mice pathway [58]. These findings provided direct evidence that miR-208a mediates TH action in heart.

### MiR-208b and miR-499

Besides miR-208a, miR-208 family contains another two miRNAs, miR-208b and miR-499. They are encoded in different myosin genes, *Myh7* (*β*-*MHC*) and *Myh7b*, respectively [60, 61]. These two intronic miRNAs have been shown to be involved in the control of muscle fiber type by activating slow and repressing fast myofiber gene programs [62]. Recently, it was reported that miR-208b and miR-499 are downstream targets of estrogen-related receptor γ (ERRγ), which mediates the effect of peroxisome proliferators-activated receptors β/δ and PPARα on energy metabolism and skeletal muscle fiber specificity [63, 64]. As a result, activation of ERRγ could lead to an upregulation of miR-208b/miR-499 and subsequently increases type I muscle fiber proportion in skeletal muscle [65]. As mentioned early, it is known that skeletal muscle is a target for TH and all members of the MyHC family respond to TH. Given that TH regulates myofiber type transition [66], it is not surprising to find that hypothyroid state induces both Myh7/miR-208b and Myh7b/miR-499 expression in skeletal muscle [62]. These findings indicate that miR-208b and miR-499 might mediate the effect of TH on myofiber type determination and energy metabolism.

### MiR-133

MiR-133 family is one of the most studied miRNA families in skeletal muscle [67]. The enrichment of miR-133 as well as miR-1 and miR-206 in heart and skeletal muscle was first reported in 2004 [68], which was confirmed subsequently by several groups. Due to their tissue specific expression, these miRNAs are designated as canonical myomiRs, which have been extensively investigated [67, 69–72]. The miR-133 family (including miR-133a1, miR-133a and miR-133b) and miR-1 family (including miR-1-1, miR-1-2 and miR-206) are clustered on human chromosomes. Their sequences and expressions are evolutionarily conserved across species [73].

Further study showed that miR-133a is highly expressed both in heart and in skeletal muscle, while miR-133b is specifically expressed in skeletal muscle [74]. Several studies confirmed that miR-133a played a regulatory role in the development of cardiac and skeletal muscle [72, 75, 76]. The most direct evidence came from miR-133a1 and miR-133a2 knockout mouse models. In the heart, absence of miR-133a resulted in ectopic expression of smooth muscle genes and aberrant cardiomyocyte proliferation due to elevated expression of SRF and cyclin D2, which were direct targets of miR-133a [72]. In the skeletal muscle, miR-133a knockout mice developed adult-onset centro nuclear myopathy in type II (fast-twitch) myofiber, accompanied by fast-to-slow myofiber transition, suggesting an essential role of miR-133a in maintaining the structure, function, and myofiber types of skeletal muscle [77].

The link between TH and myomiRs was reported in flounder (*Paralichthys olivaceus*), which showed that miR-133a as well as miR-1 and miR-206a were TH-regulated miRNAs during larval development [78]. By using hypo- and hyperthyroid mouse models and TR isoform–specific knockout mice, we demonstrated that miR-133a1 transcription could be up-regulated directly by TH in a TR-dependent manner [38]. Our study also showed for the first that overexpression of miR-133a was able to promote slow-to-fast muscle switch by repressing TEA domain family member 1 (TEAD1), a key modulator of slow muscle gene [38]. Based on our in vivo and in vitro data, we proposed that miR-133a1 mediates the effect of TH on muscle fiber type specification [38]. Furthermore, our data showed that the negative regulation of MyHC-I transcription by TH was indirect, suggesting that TH might not regulate MyHC-I transcription through a nTRE [38]. In addition, since miR-133a not only regulates myofiber type switch but also plays a role in proliferation, regeneration and remodeling, we speculated that TH signaling might have a variety of effects on muscle physiology and pathology through miR-133a1-mediated mechanisms.

### Other miRNAs

Besides myomiRs, a few other miRNAs have been shown to serve as either regulators or components of TH signaling. For example, miR-27a was demonstrated to modulate β-MHC gene expression in cardiomyocytes [79]. In cardiac hypertrophy and skeletal muscle atrophy, miR-27a was strongly increased via Srf and Myf6 transactivation [80, 81]. MiR-27a was upregulated during ES cell differentiation and heart development in mouse, implying a critical role of miR-27a in heart development [79]. Moreover, miR-27a overexpression strongly upregulated the β-MHC, but not α-MHC by targeting TRβ1 [79]. These studies highlighted the role of miR-27a in cardiac pathophysiology as a regulator in the TH signaling pathway.

MiR-30a belongs to the miR-30 family, which is highly expressed in the heart [82]. Previous studies have shown that members of miR-30 family were downregulated in cardiomyocytes in response to ROS [83]. And they also play critical roles in calcium/calcineurin signaling in cardiomyocytes, which was demonstrated by delivering a miR-30 sponge to cardiomyocytes [84]. A recent study revealed that T3 could improve the recovery of post-ischemic cardiac performance via regulating the miR-30a/p53 axis [85]. In the ischemia/reperfusion (I/R) injury model, T3 could restore the expression level of miR-30a and then prevent the upregulation of p53, suggesting an important role of miR-30a in mediating the cardioprotective effects of T3 [85].

Type 3 deiodinase (Dio3) is a TH-inactivating enzyme usually expressed at fetal stage, which regulates proliferation. In a myocardial infarction (MI) mouse model, Dio3 is up-regulated in cardiomyocytes to create a local hypothyroid condition to increase the regenerative capacity by initiating the fetal gene program. A group of miRNAs with altered expression levels were identified in this MI model and were predicted to be involved in the regulation of stress response in cardiomyocytes [86]. Further study showed that Dio3 is a target of miR-214 [87]. Therefore, the upregulation of miR-214 observed in MI model might dampen the MI-induced upregulation of Dio3. The finding that miR-214 was negatively regulated by TH suggests that miR-214 and Dio3 form a negative feedback loop in cardiomyocytes. The interplay between miR-214 and Dio3 after MI provides a mechanism to protect the adult cardiomyocyte from the adverse effect of local TH deficiency [87].

It is known that hyperthyroidism induces cardiac hypertrophy and the Angiotensin type 1 receptor (AT1R) has been demonstrated to mediate part of this response. It was shown that T3 treatment increased AT1R mRNA and protein levels rapidly in cardiomyocyte, which was accompanied with an increased of miR-350 expression. Since AT1R is one of predicted target of miR-350, it is reasonable to make the assumption that miR-350 might be involved in TH-induced cardiomyocyte hypertrophy [88]. On the other hand, two studies indicate that AT1R might contribute to the altered expression of cardiac miR-208a and miR-133 induced by hyperthyroidism [89, 90]. These findings provide new insights in the understanding of regulatory networks involved in the cardiac growth controlled by miRNAs and TH signaling.

## Perspective

**Fig. 1:**
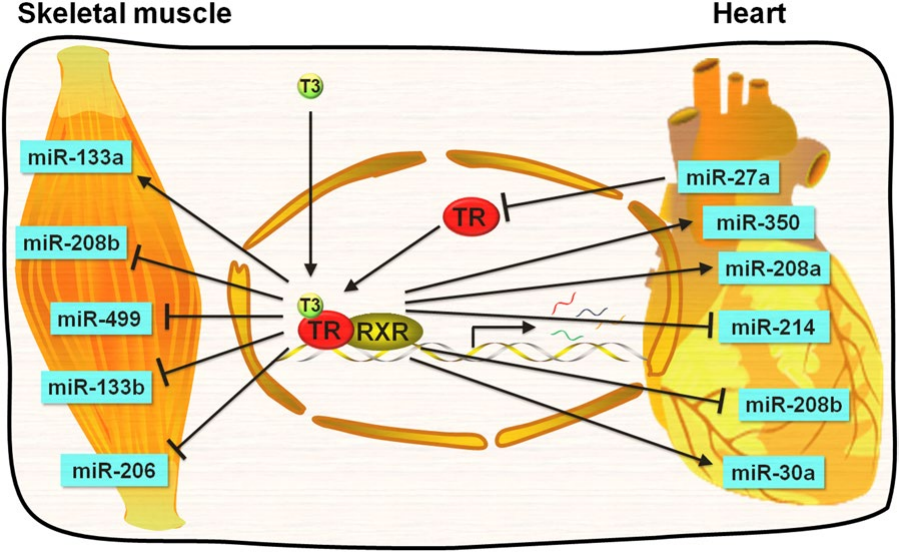
miRNAs mediates thyroid hormone action in cardiac and skeletal muscle

The effects of TH on heart and muscle have been well established. The emergence of the miRNA field has provided a unique avenue towards deeper understanding of the TH action in cardiac and skeletal muscle development, regeneration and physiology. Recent studies involving miRNAs and TH signaling in cardiac and skeletal muscle have shed light on the contributions of miRNAs to TH signaling (Fig. 1). These studies suggest that miRNAs have added an additional layer of complexity to the extensive TH effects in normal and disease states. Moreover, certain miRNAs have been proved to be the missing links to previously unrecognized mechanisms. So far, since most studies have tried to pinpoint the mechanism of miRNAs to their single downstream targets, systems biology approaches might be a better choice to explain miRNA activity under euthyroid, hyper and hypothyroid conditions. Despite the uncertainties, some of these miRNAs have the potential to eventually become heart or muscle disease biomarkers or even drug targets in future. Further investigations are required to address the relative contributions of miRNAs under physiological and pathological conditions and how a group of miRNAs are coordinated to integrate into the complex hierarchical regulatory network of TH.

## Abbreviations

TH: thyroid hormone
miRNAs: microRNAs
MyomiRs: muscle-specific miRNAs
T3: triiodothyronine
T4: thyroxine
TR: thyroid hormone receptor
TRE: thyroid hormone response element
RXR: retinoid X receptor
ATP: adenosine triphosphate
MHC: myosin heavy chain
nTRE: negative TRE
SERCA2: sarcoplasmic reticulum calcium ATPase
DRs: direct repeats
IPs: inverted palindromes
Kv: voltage-gated potassium
HCN: hyperpolarization activated cyclic nucleotide-gated
pri-miRNAs: primary miRNAs
pre-miRNAs: precursor miRNAs
RISC: RNA induced silencing complex
miRISC: miRNA-induced silencing complex
AGO2: argonaute RISC catalytic component 2
PTU: propylthiouracil
ERRγ: estrogen-related receptor γ
TEAD1: TEA domain family member 1
I/R: ischemia/reperfusion
Dio3: type 3 deiodinase
MI: myocardial infarction
AT1R: Angiotensin type 1 receptor
